# Development and internal–external cross-validation of a patient-reported definition for acute calcium pyrophosphate crystal arthritis

**DOI:** 10.1093/rheumatology/keae681

**Published:** 2024-12-14

**Authors:** Edoardo Cipolletta, Davide Rozza, Mariano Andres, Sébastien Ottaviani, Tristan Pascart, Enrique Calvo-Aranda, Maria Victoria Chiarvetto Peralta, Pietro Muto, Irene Calabuig, Silvia Gómez-Sabater, Rocío Caño, Bastien Léger, Aurore Pacaud, Erica Moscioni, Caterina Bruno, Virginia Caira, Claudia Gómez-González, Javier Eduardo Rosa, Georgina Nakafero, Emilio Filippucci, Abhishek Abhishek

**Affiliations:** Rheumatology Unit, Department of Clinical and Molecular Sciences, Polytechnic University of Marche, Ancona, Italy; Academic Rheumatology, University of Nottingham, Nottingham, UK; Epidemiology Research Unit, Italian Society of Rheumatology, Milan, Italy; Research Centre on Public Health (CESP), University of Milano-Bicocca, Monza, Italy; Department of Rheumatology, Dr Balmis General University Hospital-ISABIAL, Miguel Hernandez University, Alicante, Spain; Department of Rheumatology, Hôpital Bichat APHP Paris Nord and Université de Paris, Paris, France; Department of Rheumatology, Saint-Philibert Hospital, Lille Catholic University, Lille, France; Rheumatology Department, Hospital Universitario Infanta Leonor, Universidad Complutense de Madrid, Madrid, Spain; Hospital Italiano de Buenos Aires, Buenos Aires, Argentina; Department of Internal Medicine, Ospedale San Francesco di Paola, Cosenza, Italy; Department of Rheumatology, Dr Balmis General University Hospital-ISABIAL, Miguel Hernandez University, Alicante, Spain; Department of Rheumatology, Dr Balmis General University Hospital-ISABIAL, Miguel Hernandez University, Alicante, Spain; Department of Rheumatology, Dr Balmis General University Hospital-ISABIAL, Miguel Hernandez University, Alicante, Spain; Department of Rheumatology, Hôpital Bichat APHP Paris Nord and Université de Paris, Paris, France; Department of Rheumatology, Saint-Philibert Hospital, Lille Catholic University, Lille, France; Rheumatology Unit, Department of Clinical and Molecular Sciences, Polytechnic University of Marche, Ancona, Italy; Rheumatology Unit, Ospedale Pugliese-Ciaccio, Catanzaro, Italy; Rheumatology Unit, Ospedale Ferrari, Castrovillari, Italy; Rheumatology Department, Hospital Universitario Infanta Leonor, Universidad Complutense de Madrid, Madrid, Spain; Hospital Italiano de Buenos Aires, Buenos Aires, Argentina; Academic Rheumatology, University of Nottingham, Nottingham, UK; Rheumatology Unit, Department of Clinical and Molecular Sciences, Polytechnic University of Marche, Ancona, Italy; Academic Rheumatology, University of Nottingham, Nottingham, UK

**Keywords:** calcium pyrophosphate deposition disease, CPPD, pseudogout, chrondrocalcinosis, acute CPP crystal inflammatory arthritis

## Abstract

**Objective:**

To develop and validate a patient-reported definition of acute calcium pyrophosphate (CPP) crystal arthritis in people with crystal-proven CPP deposition (CPPD) disease.

**Methods:**

Consecutive patients with crystal-proven CPPD disease from seven centres across four countries were enrolled in a cross-sectional study. In each centre, patient-reported outcomes on the features of acute CPP crystal arthritis were collected. The expert opinion of an independent rheumatologist was the reference standard. We developed definitions based on multivariable logistic regression model with backward selection of predictors and classification and regression tree (CART) approaches.

**Results:**

Two hundred and forty-six patients [mean age 73.2 years (s.d. 10.7), 65.9% female] were enrolled. At the time of the assessment, acute CPP crystal arthritis was diagnosed in 96/246 (39.0%) participants.

Patient-reported joint warmth, patient-reported joint swelling, time from pain onset to peak, and self-reported acute CPP crystal inflammatory arthritis were included in the multivariable logistic model. This model had good discrimination (optimism-adjusted c-index: 0.92; 95% CI: 0.89, 0.95) and calibration (optimism-adjusted calibration slope: 0.95; 95% CI: 0.71, 1.19; optimism-adjusted calibration-in-the-large: 0.005; 95% CI: −0.37, 0.37) in the internal validation. Probability threshold ≥0.53 had sensitivity of 0.83 (95% CI: 0.74, 0.90) and specificity of 0.86 (95% CI: 0.79, 0.91). Performances were similar in the internal–external cross-validation. The CART identified patient-reported acute CPP crystal inflammatory arthritis, followed by joint swelling and joint warmth as the most informative variables for ascertaining acute CPP crystal arthritis [sensitivity 0.83 (95% CI: 0.72, 0.91) and specificity 0.83 (95% CI: 0.74, 0.90)].

**Conclusion:**

We developed and initially validated easy-to-use patient-reported definitions for acute CPP crystal arthritis for use in clinical trials and observational research in CPPD.

Rheumatology key messages

- We developed and initially validated easy-to-use patient-reported definitions for acute CPP crystal arthritis.
- Patient-reported joint warmth, patient-reported joint swelling, time from pain onset to peak <48 h and self-reported acute CPP crystal inflammatory arthritis were included in the definition.

## Introduction

In recent years, calcium pyrophosphate (CPP) deposition (CPPD) disease has stepped out from the margins of medical research [1]. In 2022, the first consensus-based definitions for imaging features of CPPD were published [2]. In 2023, an American College of Rheumatology (ACR)/European Alliance of Associations for Rheumatology (EULAR) task force developed and validated the first classification criteria for CPPD disease [3, 4]. Acute CPP crystal arthritis is by far the most well recognized phenotype of CPPD disease. It was the most prevalent clinical presentation reported in the international cohort of patients assembled to develop and validate the ACR/EULAR classification criteria for CPPD disease [5]. A quarter of patients experienced recurrent episodes of acute CPP crystal inflammatory arthritis during a mean follow-up of 5 years [6].

Although interventional [7] and observational [8–13] studies have evaluated the short-term efficacy of different drugs in acute CPP crystal arthritis, there is no high-quality evidence evaluating their effectiveness in preventing recurrences [14, 15].

One of the issues hampering further research in this field is the lack of validated definitions for clinical states in CPPD disease and monitoring treatment responses [16]. Research in gout faced a similar issue before the development of patient-reported definitions for gout flares in 2012 [17]. These definitions have been used in several observation studies and clinical trials to enable a standardized assessment of gout flares.

The diagnosis of acute CPP crystal arthritis can be even more challenging than that of gout flares, and a reliable self-report definition of flares is needed to support future trials and observational research in the field. Therefore, we aimed to develop and validate a patient-reported definition of acute CPP crystal arthritis. The development of a definition for acute CPP crystal arthritis may be the first step to create a broader set of response/remission criteria for CPPD disease.

## Methods

### Study design and patients

This was a multicentre cross-sectional study carried out at seven institutions across four countries (Argentina, France, Italy and Spain) between September 2022 and December 2023. We enrolled consecutive patients with a crystal-proven diagnosis of CPPD disease during routine or urgent inpatient and outpatient clinical care regardless of their clinical phenotype (e.g. acute CPP crystal arthritis, osteoarthritis with CPPD disease, chronic CPP crystal inflammatory arthritis) and reasons for their medical appointment (e.g. first presentation or follow-up assessment). We excluded patients with other known inflammatory arthritis (e.g. gout, rheumatoid arthritis).

At each institution, two investigators independently evaluated each participant: a study investigator and a rheumatologist with expertise in CPPD diagnosis and care (i.e. the diagnostic standard investigator).

Study investigators collected clinical and demographic data and administered a standardized questionnaire. They were instructed not to disclose their clinical judgement with either participants or diagnostic standard investigators before their participants’ assessment to avoid information bias.

Diagnostic standard investigators were asked to decide whether the participant was having acute CPP crystal arthritis. To avoid any circular reasoning, they could perform a clinical assessment and evaluate laboratory results and imaging findings. They also had full access to pain anamnesis but they were blinded to the responses contained in the patient’s questionnaire.

An investigator (E.C.) from the coordinating centre (Polytechnic University of Marche, Italy) trained the investigators of each centre to achieve a standardized patient assessment across the sites before the start of the study.

### Data collection

Study investigators collected the following data: age, sex, ethnicity, aetiology of CPPD disease (i.e. idiopathic or secondary to metabolic, endocrine or genetic disorders), presence of tenderness and swelling using the 66/68 tender/swollen joints count, duration since the diagnosis, medications and comorbidities. When available, results of inflammatory markers (i.e. erythrocyte sedimentation rate and C-reactive protein) and synovial fluid analysis (i.e. leucocyte count and presence of CPP crystals) performed on the same date of or within one week before the clinical assessment were collected.

Each participant self-identified the ‘target joint’ as the most inflamed joint. Study investigators then administered a standardized questionnaire, developed based on the 2012 provisional gout flare definition and an OMERACT systematic literature review on the outcome measures in acute CPP crystal arthritis [17–19]. It contains the following questions:

- Patient self-reported acute CPP crystal arthritis: ‘Acute CPPD disease flare can be described as a sudden onset of severe joint pain, swelling and an inability to bear weight or to mobilize the affected joint were common symptoms. These manifestations are usually abrupt, alarming, or severe that cause the majority of patients to seek medical attention [20]. Are you having an acute CPP crystal arthritis today?’ Yes/No.
- Patient self-reported joint swelling: ‘Are any of your joints swollen?’ Yes/No.
- Patient self-reported joint warmth: ‘Are any of your joints warm to touch?’ Yes/No.
- Patient self-reported joint pain: ‘Are any of your joints painful?’ Yes/No.
- Pain at rest: ‘Considering pain from your CPPD disease over the last week when you are resting (for example in bed or sitting quietly), please circle the number indicating the level of pain when it was at its worst.’ The answer will be noted on a 0–10 numeric rating scale.
- Time needed to reach peak pain intensity: ‘From the onset/worsening of pain, the pain has reached the maximum intensity within (A) ≤12 h, (B) 13–48 h, (C) >48 h.’

In addition, the study investigator administered the health assessment questionnaire-II (HAQ) [21].

We considered these seven candidate predictors to develop the patient-reported definitions. A standardized questionnaire was made available in English to all study centres. Questionnaires were translated into the local study site language by the site investigators or a professional medical translator with the final approval of the investigators.

### Definition of acute CPP crystal arthritis

Similar to other diagnostic and classification criteria in rheumatology [3, 22], the clinical judgement of expert rheumatologists (i.e. diagnostic standard investigators) was the reference standard in the main analysis.

Diagnostic standard investigators were asked the question ‘Do you believe the participant is having an acute CPP crystal arthritis today?’ Yes/No.

In addition, the following data were recorded:

- Physician-reported joint tenderness. Yes/No.
- Physician-reported joint swelling. Yes/No.
- Physician-reported joint warmth. Yes/No.

### Statistical analysis

We developed patient-reported definitions based on a multivariable regression model [23] and a classification and regression tree (CART) analysis [24].

To describe participants’ characteristics, we employed descriptive statistics: absolute and relative frequencies for categorical variables, mean and standard deviation for continuous ones. The concordance between physician- and patient-reported outcomes was measured using total agreement and unweighted Cohen’s kappa. We used STATA 18 (StataCorp, College Station, TX, USA) to analyse the data. We wrote this article in accordance with the TRIPOD statement [25].

#### Multivariable logistic regression model

##### Model development

To allow for maximum simplification of the variables included in the models, we compared the discriminative ability of the time needed to reach peak pain intensity considered as a binary or as an ordinal variable. Since both the categories ≤12 h and 13–48 h were significantly associated with the reference standard with comparable strength of association, the variable was handled as a binary variable (i.e. ≤48 h *vs* >48 h) (Supplementary Data S1, available at *Rheumatology* online).

A backward elimination procedure (*P* for removal >0.10) starting from a multivariable logistic regression model conditioned on all the patient-reported outcomes included in the questionnaire was used to identify the variables independently associated with the reference standard. We modelled continuous predictors (i.e. pain at rest and HAQ) using first-order fractional polynomials, with functional forms chosen in the presence of all candidate predictors. Transformed terms were not better than the linear terms (*P* > 0.05), and hence they were not transformed.

We checked for specification errors [we used the linear predicted value (_hat) and linear predicted value squared (_hatsq) as the predictors to rebuild the model using the ‘linktest’ command] and collinearity issues (we used tolerance and variance inflation factor using the ‘collin’ command).

##### Internal validation

Internal validation was conducted using bootstrapping with 1000 samples with replacement from the whole dataset. The predictive performance of the model developed in each bootstrap was assessed in both that sample and the original dataset, to gain estimates of optimism (average difference in the performance in the original dataset and the bootstrap samples), and model performance estimates were adjusted accordingly using a uniform shrinkage factor. The original β-coefficients were multiplied by the uniform shrinkage factor and the intercept re-estimated conditional on the shrunken β-coefficients to ensure that overall calibration was maintained, producing a final model after internal validation.

The performance of the models was reported as discrimination and calibration. Discrimination was measured using sensitivity, specificity and the c-index—which is equivalent to the area under the receiver operating characteristic (ROC) curve—with their 95% CI. Calibration was quantified using the calibration slope (where 1.00 is the ideal) and the calibration-in-the-large (CITL) (where 0 is the ideal).

Finally, we plotted a ROC curve of our final diagnostic model after internal validation and selected the point of maximum discriminating ability as the threshold for diagnosing acute CPP crystal arthritis.

##### Internal–external cross-validation

We used an internal–external cross-validation approach for further validating the proposed definitions in the development dataset, across subgroups by participating centres. In each cycle, the model development and internal validation process (as described above) was repeated using all but one of the groups. This model was then applied to the omitted data, and its predictive performance was assessed. Following all cycles, performance estimates were summarized using a random-effects meta-analysis estimated using restricted maximum likelihood and the Hartung–Knapp–Sidik–Jonkman variance correction for CITL, c-index and c-slope, and using a hierarchical summary ROC model for sensitivity and specificity (STATA command metadta). The internal–external cross-validation measures the heterogeneity in performance of the model across centres and provides initial evidence on the validity and generalizability of the model [26].

#### CART analysis

CART is a machine learning classification and regression algorithm that helps to identify the most predictive variables based on their informativeness [24]. CART analysis is a non-parametric form of binary recursive partitioning. Therefore, no assumptions are made regarding the underlying distribution of values of the predictor variables.

CART analysis consists of four basic steps. The first step consists of tree building, during which a tree is built by selecting the most informative split among the variables at each node. The second step consists of stopping the tree-building process. At this point, a ‘maximal’ tree has been produced, which probably greatly overfits the information contained within the learning dataset. The third step consists of tree ‘pruning’, which results in the creation of a sequence of simpler and simpler trees, through the removal of less important terminal nodes. Stopping rules can also be implemented to control the depth of the tree. The fourth step consists of optimal tree selection, during which the tree that best fits the information in the learning dataset, but does not overfit the information, is selected.

In this study, we developed an honest classification tree using the one standard error rule, no stopping rules, the Gini index as the measure of impurity, and a 3-fold cross-validation to avoid the overfitting of the model. The honest tree in this classification case minimizes an error-complexity function given by the sum of the tree’s (*T*) misclassification error and its standard error:


$$R\left(T\right)+s\times \mathrm{SE}\left(R\left(i\right)\right)$$

with *s* being a tuning parameter. The simplest tree satisfying this condition is chosen through this procedure. We used the STATA package ‘crtrees’ for the CART analysis.

#### Sensitivity analyses

We performed a set of sensitivity analyses to test the robustness of the proposed definitions. We (i) stratified the analyses on the history of previous acute CPP crystal arthritis (yes *vs* no), sex (female *vs* male), and the target joint (knee *vs* other joints); (ii) excluded one enrolling centre at a time to evaluate whether removing participants from that site did not significantly change the diagnostic accuracy of the proposed criteria; (iii) replaced patient-reported joint swelling and patient-reported joint warmth with the corresponding physician-assessed measures; (iv) added blood inflammatory markers among the variables included in the analyses; and (v) included pain at rest numeric rating scale >1 to >5 as an absolute inclusion criterion for having acute CPP crystal arthritis.

#### Sample size

Using recommendations to minimize overfitting [27], the minimal sample size required for model development was 246 participants with 50 events, based on anticipated seven predictor parameters, Cox–Snell *R*^2^ of 0.30, outcome prevalence of 0.20 and uniform shrinkage factor of 0.90.

## Results

### Patients

We enrolled 246 patients [mean age was 73.2 years (s.d. 10.7 years), 65.9% female] (Table 1). At the time of the assessment, 96/246 (39.0%) participants had acute CPP crystal arthritis, 39 (15.9%) had chronic inflammatory CPP crystal arthritis and 111 (45.1%) OA+CPPD disease according to the judgement of the diagnostic standard investigators who acted as the reference standard. SF was available in 73/246 (29.7%) participants, and 41/73 (56.2%) had a leucocyte count >2000 cells/μl and CPP crystals in the SF.

**Table 1.** Baseline clinical and demographic characteristics of the enrolled patients

	Physician-defined acute CPP crystal arthritis		
	Total	No	Yes
	(*n* = 246)	(*n* = 150)	(*n* = 96)
Age, mean (s.d.), years	73.2 (10.7)	73.0 (10.1)	73.5 (11.5)
Sex, female, *n* (%)	162 (65.9)	103 (68.7)	59 (61.5)
BMI, mean (s.d.), kg/m^2^	27.1 (4.7)	27.8 (5.0)	26.0 (3.9)
Ethnicity, Caucasian, *n* (%)	243 (98.8)	148 (98.7)	95 (99.0)
CPPD disease aetiology			
Idiopathic, *n* (%)	235 (95.5)	143 (95.3)	92 (95.8)
Secondary to metabolic/endocrine/genetic disorders, *n* (%)	11 (4.5)	7 (4.7)	4 (4.2)
History of previous episodes of acute CPP crystal arthritis, *n* (%)	205 (83.3)	126 (84.0)	79 (82.2)
Increased serological inflammatory markers, *n* (%)	88 (44.9)	37 (30.6)	51 (68.0)
Missing data, *n* (%)	50 (20.3)	29 (19.3)	21 (21.9)
SFA with CPP crystals + leucocyte count >2000 cells/μl, *n* (%)	36 (49.3)	2 (6.3)	41 (42.7)
Missing data, *n* (%)	173 (70.3)	118 (78.7)	34 (82.9)
Target joint			
Shoulder, *n* (%)	22 (8.9)	15 (10.0)	7 (7.3)
Elbow, *n* (%)	5 (2.0)	2 (1.3)	3 (3.1)
Wrist, *n* (%)	57 (23.2)	31 (20.7)	26 (27.1)
Small joints of hands and feet, *n* (%)	12 (4.9)	9 (6.0)	3 (3.1)
Hip, *n* (%)	6 (2.4)	4 (2.7)	2 (2.1)
Knees, *n* (%)	119 (48.4)	72 (48.0)	47 (49.0)
Ankle, *n* (%)	25 (10.2)	17 (11.3)	8 (8.3)
Enrolling centres			
Alicante, Spain, *n* (%)	25 (14.2)	33 (22.0)	2 (2.1)
Ancona, Italy, *n* (%)	50 (20.3)	31 (20.7)	19 (19.8)
Buenos Aires, Argentina, *n* (%)	27 (11.0)	5 (3.3)	22 (22.9)
Cosenza/Catanzaro, Italy, *n* (%)	47 (19.1)	27 (18.0)	20 (20.8)
Lille, France, *n* (%)	28 (11.4)	18 (12.0)	10 (10.4)
Madrid, Spain, *n* (%)	31 (12.6)	22 (14.7)	9 (9.4)
Paris, France, *n* (%)	28 (11.0)	14 (9.3)	14 (14.6)
Physician-reported outcomes			
Physician-reported joint tenderness, *n* (%)	195 (79.3)	100 (66.7)	95 (99.0)
Physician-reported joint swelling, *n* (%)	143 (58.1)	57 (38.0)	86 (89.6)
Physician-reported joint warmth, *n* (%)	76 (30.9)	16 (10.7)	60 (62.5)
Patient-reported outcomes			
Patient-reported joint tenderness, *n* (%)	185 (75.2)	103 (68.7)	82 (85.4)
Patient-reported joint swelling, *n* (%)	138 (56.1)	53 (35.3)	85 (88.5)
Patient-reported joint warmth, *n* (%)	116 (47.2)	44 (29.3)	72 (75.0)
Time needed to reach peak pain intensity			
≤12 h, *n* (%)	56 (22.8)	24 (16.0)	32 (33.3)
13–48 h, *n* (%)	69 (28.0)	29 (19.3)	40 (41.7)
>48 h, *n* (%)	121 (49.2)	97 (64.7)	24 (25.0)
Patient-reported acute CPP crystal arthritis, *n* (%)	120 (48.8)	38 (25.3)	82 (85.4)
Pain at rest, mean (s.d.), 0–10 NRS	5.0 (2.9)	3.5 (2.9)	6.8 (2.8)
HAQ, mean (s.d.)	1.0 (0.6)	1.0 (0.6)	1.1 (0.6)

CPP: calcium pyrophosphate; CPPD: calcium pyrophosphate deposition; HAQ: health assessment questionnaire; NRS: numeric rating scale; SFA: synovial fluid analysis.

### Agreement between physician- and patient-defined outcomes

The concordance between diagnostic standard investigators and participants in defining acute CPP crystal arthritis was 78.9% (*n*/*N*: 194/246); kappa: 0.58 (95% CI: 0.47, 0.68). In 38 cases (15.4%), participants considered that they had acute CPP crystal arthritis, while diagnostic standard investigators disagreed. In 14 (5.7%) cases, the opposite scenario occurred.

The agreement between the judgement of diagnostic standard investigators and the results of the synovial fluid analysis (i.e. presence of a leucocyte count >2000 cell/μl + presence of CPP crystals) was 87.7% (*n*/*N*: 64/73); kappa: 0.75 (95% CI: 0.61, 0.90). In seven cases (9.6%), diagnostic standard investigators considered that a participant had acute CPP crystal arthritis, while synovial fluid analysis did not show a leucocyte count ≥2000 cells/μl. In two (2.7%) cases, the opposite scenario occurred.

### Multivariable logistic regression model

Patient-reported acute CPP crystal arthritis, patient-reported swelling of a joint, time needed to reach the peak pain intensity (<48 h), and patient-reported warmth of a joint were associated with a diagnosis of acute CPP crystal arthritis according to the judgement of diagnostic standard investigators, and they were retained in the final multivariable model (Supplementary Data S2, available at *Rheumatology* online). We did not detect any collinearity among the selected variables.

Original, apparent and optimism-adjusted performance of the model in the development dataset are reported in Table 2. Before shrinkage (original performance), the calibration slope, the CITL and the c-index in the development data were 1.00 (95% CI: 0.76, 1.24), 0.00 (95% CI: −0.38, 0.38) and 0.92 (95% CI: 0.89, 0.95), respectively. From the bootstrap, a uniform shrinkage factor of 0.95 was obtained and used to shrink predictor coefficients in the final model to account for optimism and to re-estimate the final model’s intercept. The optimism-adjusted calibration slope, CITL and c-index after internal validation were 0.95 (95% CI: 0.71, 1.19), −0.005 (95% CI: −0.37, 0.37) and 0.92 (95% CI: 0.89, 0.95), respectively.

**Table 2.** Diagnostic performance for the proposed patient-reported definitions for acute CPP crystal arthritis developed using the multivariable logistic regression model

	Original performance	Apparent performance in the bootstrap samples	Average optimism	Optimism-adjusted performance	Internal-external cross-validation
**c-slope**	1.0 (0.76, 1.24)	0.95 (0.94, 0.96)	0.05	0.95 (0.71, 1.19)	0.97 (0.44, 1.55)
**CITL (95% CI)**	0.00 (−0.38, 0.38)	−0.005 (−0.01, 0.02)	−0.005	−0.005 (−0.37, 0.37)	0.32 (−0.95, 1.59)
**c-index (95% CI)**	0.92 (0.89, 0.95)	0.92 (0.92, 0.92)	0.01	0.92 (0.89, 0.95)	0.92 (0.85, 0.99)
**Sensitivity (95% CI)**	0.83 (0.74, 0.90)	—	—	—	0.76 (0.66, 0.84)
**Specificity (95% CI)**	0.86 (0.79, 0.91)	—	—	—	0.88 (0.75, 0.94)

CITL: calibration in the large; CPP: calcium pyrophosphate.

**Figure 1.**
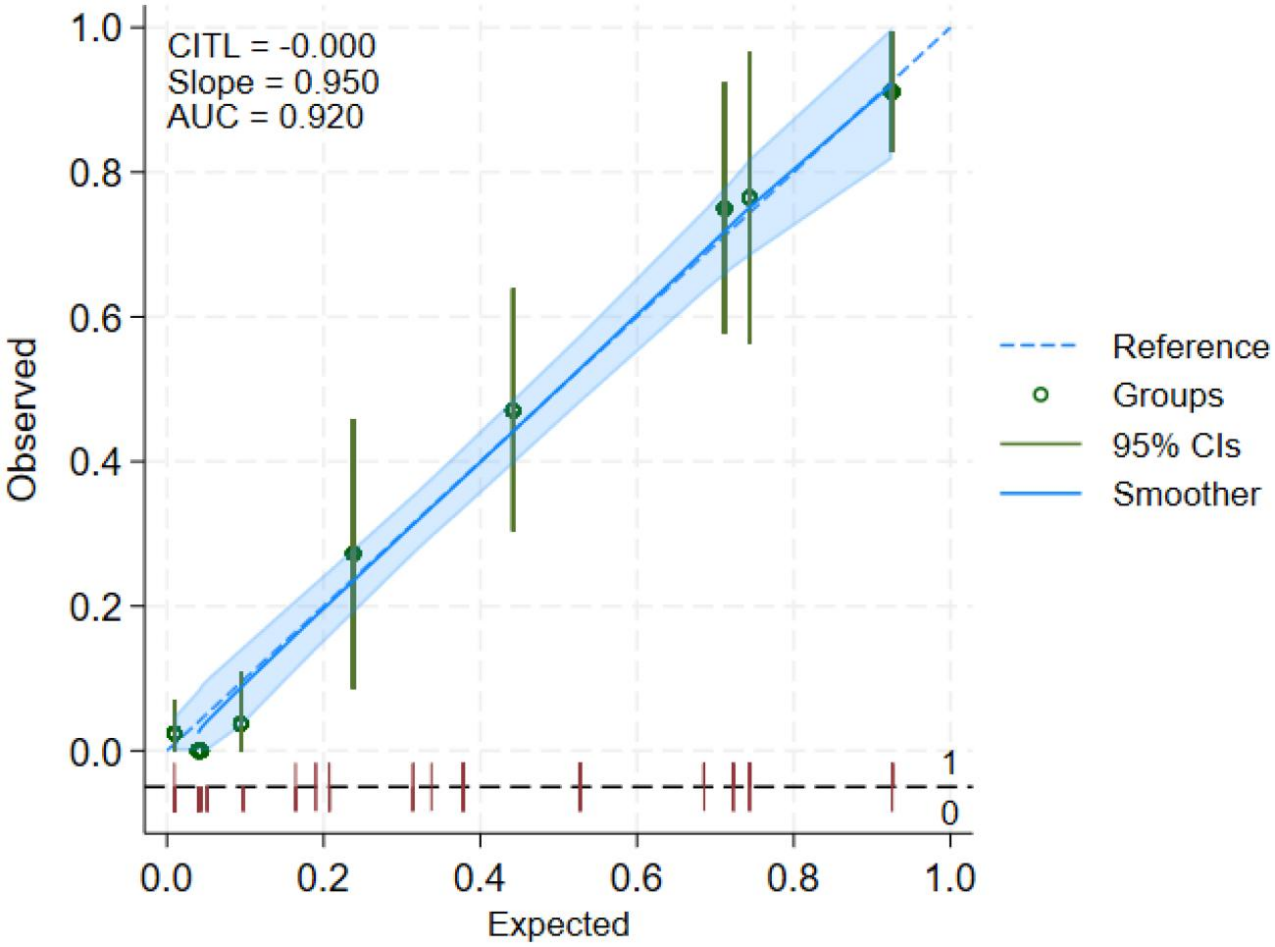
Calibration plot of the patient-reported definition of acute CPP crystal arthritis in model development data after internal validation. AUC: area under the curve; CITL: calibration in the large; CPP: calcium pyrophosphate

Figure 1 shows the calibration plot for the final model.

A probability ≥0.53 was selected as the threshold with the highest discriminative value (Supplementary Data S3, available at *Rheumatology* online). This probability corresponded to having at least three out of four criteria included in the final model (Supplementary Data S4, available at *Rheumatology* online), and therefore these two cut-off values were used interchangeably.

The pooled performance of this model in the internal–external cross-validation approach was comparable to that of the internal validation (Table 2 and Supplementary Data S5, available at *Rheumatology* online).

### Classification and regression tree model

**Figure 2.**
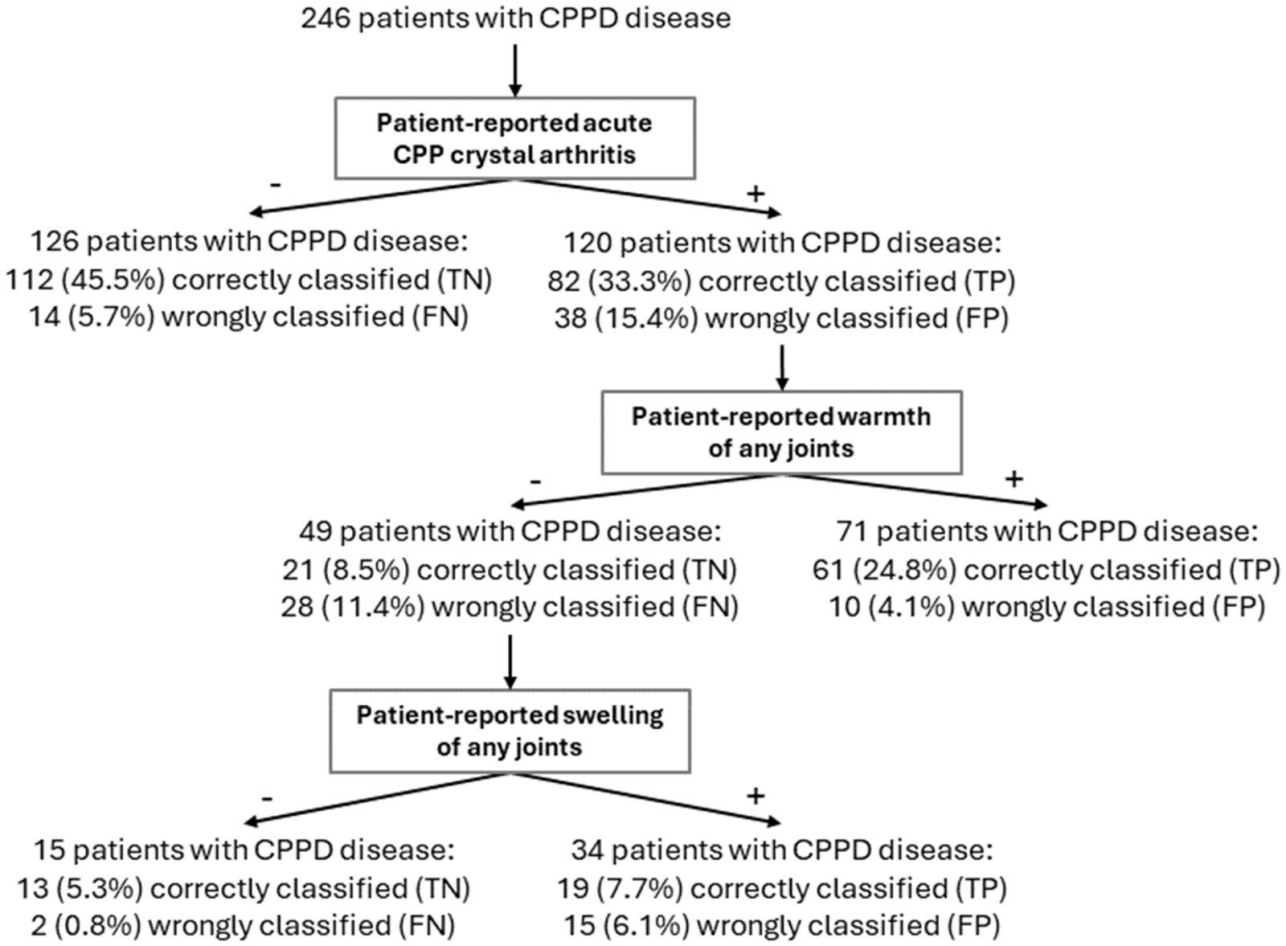
Classification tree for a patient-reported definition of acute CPP crystal arthritis in the model development set (*N* = 246). In every percentage displayed in the figure, the total number of patients included in the model development set (*N*=246) is the denominator. CPP: calcium pyrophosphate; CPPD: calcium pyrophosphate deposition; FN: false negative; FP: false positive; NPV: negative predictive value; PPV: positive predictive value; TN: true negative; TP: true positive

Of the seven variables evaluated, the CART method identified patient-reported acute CPP crystal arthritis as the best single discriminator between those diagnosed with and without acute CPP crystal arthritis by diagnostic standard investigators, followed by patient-reported joint swelling, and patient-reported joint warmth. Figure 2 depicts the final tree generated by the CART analysis in the model development set.

The definition developed using the CART method had a sensitivity of 0.83 (95% CI: 0.74, 0.90), specificity of 0.83 (95% CI: 0.76, 0.89) and a c-index of 0.83 (95% CI: 0.79, 0.88) in the model development set.

### Sensitivity analyses

The results of the sensitivity analyses were consistent with those of the main analyses (Supplementary Data S6, available at *Rheumatology* online). In particular, the definitions performed well in both participants with and participants without a history of previous episodes of acute CPP crystal arthritis. Also, the discrimination of these definitions was quite homogeneous across centres (Supplementary Data S7, available at *Rheumatology* online). In addition, the performance of these definitions was not significantly improved by the addition of raised blood inflammatory markers nor when we replaced patient-reported with physician-based outcomes.

Table 3 and Supplementary Data S8, available at *Rheumatology* online, summarize the proposed patient-reported definitions for acute CPP crystal arthritis.

**Table 3.** Proposed patient-reported definitions for acute CPP crystal arthritis

Domain	Question	Answer
Patient self-reported acute CPP crystal arthritis	Acute CPPD flare can be described as a sudden onset of severe joint pain, swelling and an inability to bear weight or to mobilize the affected joint were common symptoms. These manifestations are usually abrupt, alarming or severe that cause the majority of patients to seek medical attention. Are you having an acute CPP crystal arthritis today?	No/Yes
Patient self-reported joint swelling	Are any of your joints swollen?	No/Yes
Patient self-reported joint warmth	Are any of your joints warm to touch?	No/Yes
Time needed to reach peak pain intensity	From the onset/worsening of pain, has the pain reached the maximum intensity within ≤48 h?	No/Yes
**Definitions based on multivariable logistic model**		
≥3 positive answers classify the patient as having acute CPP crystal arthritis		
**Definitions based on classification and regression tree analysis**		
Patient self-reported acute CPP crystal arthritis *and either* patient self-reported joint swelling *or* patient self-reported joint warmth classify the patient as having acute CPP crystal arthritis		

CPP: calcium pyrophosphate; CPPD: calcium pyrophosphate deposition.

## Discussion

We developed and validated patient-reported definitions for acute CPP crystal arthritis using two different analytical approaches and the judgement of diagnostic standard investigators as the reference standard. These definitions may be used to capture the occurrence of acute CPP crystal arthritis as the outcome of interest in long-term studies of patients with CPPD disease. In addition, this definition may be included in a set of core domains for the development of response/remission criteria for CPPD disease.

Four clinical features—i.e. patient-reported acute CPP crystal arthritis, patient-reported joint swelling, patient-reported joint warmth, and the time needed to reach the peak pain intensity—were included in the definitions. All these variables have been selected from a list of potential outcomes in CPPD studies developed by the OMERACT CPPD Working Group [17, 18].

Among these four variables, patient-reported acute CPP crystal arthritis was the most informative variable followed by patient-reported joint swelling, and patient-reported joint warmth in the CART analysis.

Patients with CPPD disease are usually old and have coexistent osteoarthritis [7]. In our cohort, 75.2% of participants self-reported pain and up to 79% had tenderness of the target joint. This may explain why pain was not discriminatory enough to be included in any of the definitions for acute CPP crystal arthritis and why considering pain at rest did not improve the performance of the proposed definitions.

The definitions based on the logistic regression model were slightly more specific than the one developed using the CART methodology (0.86 *vs* 0.83). The former definitions are more flexible than the latter since the use of different cut-off values allows for increasing either the sensitivity or the specificity as required in the specific clinical context. We selected a cut-off of ≥3/4 criteria—corresponding to a probability of ≥0.53—as it has shown the best discriminative ability and the best balance between sensitivity and specificity. A further external validation study is required to define which definition should be preferred.

Our definition is easy to use. It only requires patient-reported information, an approach supported by the EULAR [28], and already used in other diseases [29, 30]. The inclusion of physical examination findings and inflammatory markers did not improve the diagnostic accuracy. Thus, this definition is not resource intensive. In addition, the accuracy of these definitions was quite homogeneous across participating centres, in patients who had or had not previous episodes of acute CPP crystal arthritis, and when the affected joint was or was not the knee. However, it should be borne in mind that >80% of participants had a previous episode of acute CPP crystal arthritis. These patients have already had experience of the disease, and therefore they may recognize a future flare more accurately compared with those experiencing acute CPP crystal arthritis for the first time.

We developed these definitions using established methodologies that have already been used by several international initiatives endorsed by the ACR and the EULAR [31–33]. The judgement of expert rheumatologists as the reference standard has also been used in previous research and classification criteria [3, 22]. We assured independent data collection and case ascertainment to avoid circular reasoning. We also enrolled patients from routine clinical practice and from different countries to improve the generalizability of the results.

This study has also some limitations. First, it is possible that some patients with chronic CPP crystal inflammatory arthritis or osteoarthritis with CPPD who do not currently have acute CPP crystal arthritis may fulfil the proposed definition for acute CPP crystal arthritis. CPPD disease is a multifaceted and complex disease in which structural damage often coexists with joint inflammation, and patients may experience overlapping disease states.

Second, a standardized physician definition for acute CPP crystal arthritis is lacking. We used the clinical judgement of expert rheumatologists as the reference standard and we tested the agreement with synovial fluid analysis where available. However, we did not perform an adjudication of each case, and therefore we cannot exclude a certain risk of misclassification. Nevertheless, we enrolled patients with a crystal-proven diagnosis of CPPD disease and we excluded patients with a known diagnosis of other inflammatory arthropathies to minimize this potential bias. On the other hand, the use of synovial fluid analysis in all cases as a more objective reference standard would have imposed ethical issues such as performing joint puncture in patients without a true clinical need.

Third, the natural history of acute CPP crystal arthritis could be significantly modified by the use of anti-inflammatory medications. In the present observational study, we did not apply any selection criteria for patients to be enrolled. Therefore, we were not able to test the performance of these definitions in patients who were either treated or not treated with anti-inflammatory drugs.

Fourth, we did not perform an external validation of these definitions. Therefore, they should be validated in future studies.

Fifth, nearly all participants reported a Caucasian ethnicity. Thus, the performance of these definitions in other ethnic groups should be tested before their use.

Sixth, the questionnaire administered to participants was not formally validated before the start of the study. However, the performance of the developed acute CPP crystal definition was comparable across centres in different countries. Therefore, we believe that it should not have led to major bias.

Seventh, some patient-reported outcomes—i.e. patient-reported joint warmth, patient-reported joint tenderness, patient-reported joint swelling and patient self-reported acute CPP crystal arthritis—were recorded as binary variables instead of using a Likert scale. We chose to do so to improve the feasibility of our questionnaire and increase patients’ acceptability, aware of the fact that CPPD disease mainly affects older people.

Eighth, we agreed on allowing the diagnostic standard investigators to have full access to patients’ data as we felt that the risk of making a wrong diagnosis was greater than the risk of circular reasoning. However, they assessed the patients after the collection of patient-reported outcomes to avoid influencing participants’ judgment and minimize the risk of circular reasoning.

Finally, as no participant had missing data on the variables included in the model, these definitions cannot be applied when patients had missing data on relevant patient-reported outcomes.

We present definitions for acute CPP crystal arthritis in patients with CPPD disease, as part of an effort to develop response criteria in CPPD research. These definitions are to be used in clinical trials and observational research. These definitions ought to be validated in other populations.

## Supplementary Material

keae681_Supplementary_Data

## Data Availability

Raw data and study protocol are available upon reasonable request from the corresponding author.
