# Supplementary material for: Development and internal–external cross-validation of a patient-reported definition for acute calcium pyrophosphate crystal arthritis
Source: Rheumatology (Oxford). 2024 Dec 14;64(5):2609–17. doi: 10.1093/rheumatology/keae681 (PMC12048045; doi:10.1093/rheumatology/keae681)
Supplement: keae681_Supplementary_Data [file keae681_supplementary_data.docx]

**Supplementary Data S1**. Multivariable logistic regression model developed using the variable “Time needed to reach peak pain intensity” as a binary variable (A) and an ordinal variable (B).

**Model A**

| **Variables** | **Coefficients** | **95% confidence interval** | | **P value** |
| --- | --- | --- | --- | --- |
|  |  | **Lower bound** | **Upper bound** |  |
| Patient self-reported acute CPP crystal arthritis (yes) | 2.70 | 1.79 | 3.62 | <0.001 |
| Patient-reported joint swelling (yes) | 1.89 | 0.98 | 2.81 | 0.001 |
| Patient-reported joint tenderness (yes) | 0.13 | -0.90 | 1.16 | 0.956 |
| Patient-reported joint warmth (yes) | 1.82 | 0.96 | 2.68 | 0.009 |
| Pain at rest - 0-10 numeric rating scale | -0.11 | -0.26 | 0.03 | 0.465 |
| Time needed to reach peak pain intensity (<48 hours) | 1.62 | 0.80 | -2.43 | 0.005 |
| Health assessment questionnaire (0-3) | 0.08 | -0.54 | 0.69 | 0.639 |
| Intercept | -3.15 | -4.54 | -1.76 | 0.001 |

**Model B**

| **Variables** | | **Coefficients** | **95% confidence interval** | | **P value** |
| --- | --- | --- | --- | --- | --- |
|  |  |  | **Lower bound** | **Upper bound** |  |
| Patient self-reported acute CPP crystal arthritis (yes) | | 2.71 | 1.79 | 3.63 | <0.001 |
| Patient-reported joint swelling (yes) | | 1.90 | 0.98 | 2.82 | 0.001 |
| Patient-reported joint tenderness (yes) | | 0.14 | -0.89 | 1.17 | 0.956 |
| Patient-reported joint warmth (yes) | | 1.85 | 0.98 | 2.72 | 0.009 |
| Pain at rest - 0-10 numeric rating scale | | -0.12 | -0.26 | 0.03 | 0.465 |
| Time needed to reach peak pain intensity | <12 hours | 1.48 | 0.48 | 2.48 | 0.005 |
|  | 13-48 hour | 1.72 | 0.78 | 2.66 | <0.001 |
|  | >48 hours | Reference | Reference | Reference | / |
| Health assessment questionnaire (0-3) | | 0.08 | -0.53 | 0.70 | 0.787 |
| Intercept | | -4.80 | -6.30 | -3.29 | 0 |

**Supplementary Data S2**. Selection of the variables included in the multivariable logistic model using a stepwise backward selection process.

|  | Coefficient | P value | 95% confidence intervals | |  |
| --- | --- | --- | --- | --- | --- |
|  |  |  | Lower bound | Upper bound | |
| Patient-reported joint warmth (yes) | 1.18 | 0.01 | 0.26 | 2.09 | |
| Patient-reported joint swelling (yes) | 1.80 | <0.01 | 0.73 | 2.89 | |
| Time needed to reach peak pain intensity (<48 hours) | 1.30 | <0.01 | 0.41 | 2.20 | |
| Patient self-reported acute CPP crystal arthritis (yes) | 2.36 | <0.01 | 1.41 | 3.31 | |
| Intercept | -4.37 | <0.01 | -5.70 | -3.03 | |

P values for the Wald test:

- p=0.96 > 0.1, thus removing Patient-reported joint tenderness
- p=0.64 > 0.1, thus removing Health Assessment Questionnaire
- p=0.39 > 0.1, thus removing Pain at rest - 0-10 numeric rating scale

**Supplementary Data S3**. ROC curve displaying the selected threshold for diagnosing acute CPP crystal arthritis.


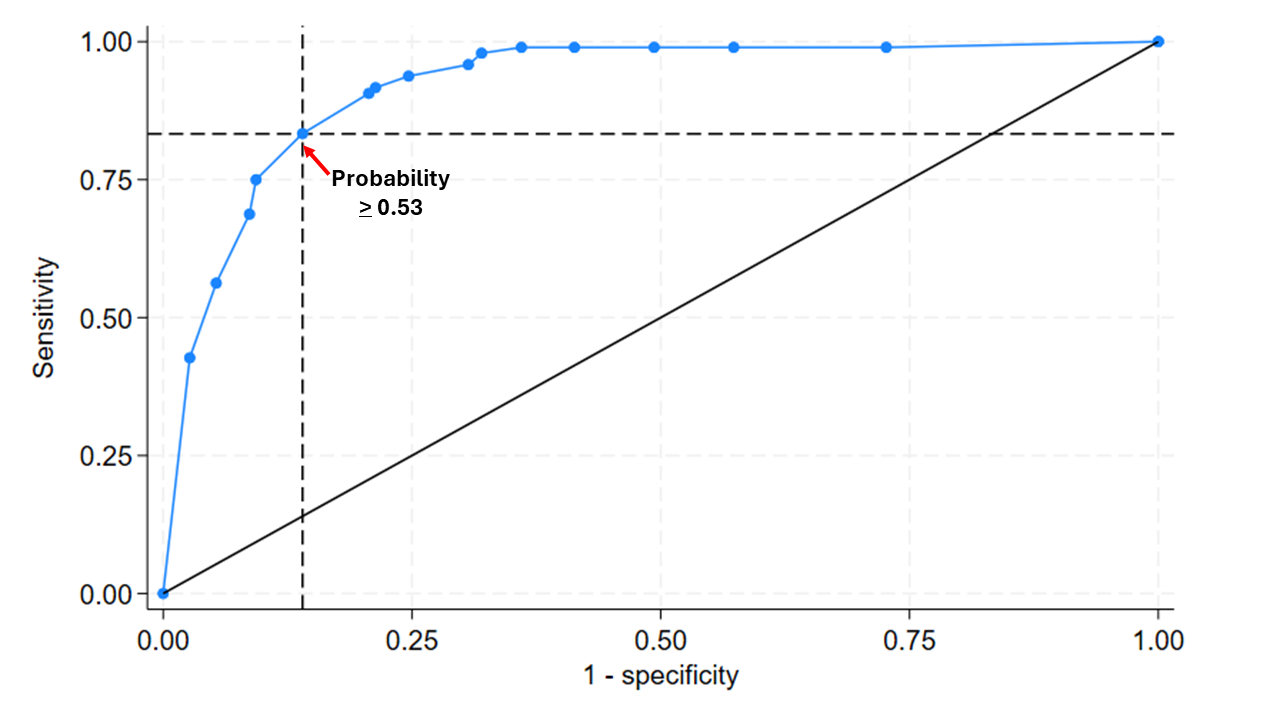


This cut-off value had the highest discriminating power between those with and without acute CPP crystal arthritis according to the expert rheumatologists as shown in the following table.

|  |  |  | Correctly |  |  |
| --- | --- | --- | --- | --- | --- |
| Cutpoint | Sensitivity | Specificity | classified | LR+ | LR- |
|  |  |  |  |  |  |
| >0.01 | 100.00% | 0.00% | 39.02% | 1 |  |
| >0.040 | 98.96% | 27.33% | 55.28% | 1.3618 | 0.0381 |
| >0.044 | 98.96% | 42.67% | 64.63% | 1.726 | 0.0244 |
| >0.05 | 98.96% | 50.67% | 69.51% | 2.0059 | 0.0206 |
| >0.10 | 98.96% | 58.67% | 74.39% | 2.3942 | 0.0178 |
| >0.16 | 98.96% | 64.00% | 77.64% | 2.7488 | 0.0163 |
| >0.19 | 97.92% | 68.00% | 79.67% | 3.0599 | 0.0306 |
| >0.21 | 95.83% | 69.33% | 79.67% | 3.125 | 0.0601 |
| >0.31 | 93.75% | 75.33% | 82.52% | 3.8007 | 0.083 |
| >0.34 | 91.67% | 78.67% | 83.74% | 4.2969 | 0.1059 |
| >0.39 | 90.62% | 79.33% | 83.74% | 4.3851 | 0.1182 |
| **>0.53** | **83.33%** | **86.00%** | **84.96%** | **5.9524** | **0.1938** |
| >0.69 | 75.00% | 90.67% | 84.55% | 8.0357 | 0.2757 |
| >0.72 | 68.75% | 91.33% | 82.52% | 7.9327 | 0.3422 |
| >0.74 | 56.25% | 94.67% | 79.67% | 10.5469 | 0.4621 |
| >0.93 | 42.71% | 97.33% | 76.02% | 16.0156 | 0.5886 |
| >0.93 | 0.00% | 100.00% | 60.98% |  | 1 |

**Supplementary Data S4**. Correspondence between the number of criteria included in the multivariable logistic regression model and the selected cut-off probability to diagnose acute CPP crystal arthritis.

| Optimism-adjusted coefficients | Criterion # | Criterion name |
| --- | --- | --- |
| -4.6417817 | / | Intercept |
| +2.4070697 | 1 | Patient-reported acute CPP crystal arthritis |
| +1.7376265 | 2 | Patient-reported swelling of a joint |
| +1.5609486 | 3 | Patient-reported warmth of a joint |
| +1.4536605 | 4 | Time needed to reach the peak pain intensity (<48 hours) |

| Number of criteria satisfied | Corresponding linear predictor | Corresponding probability |
| --- | --- | --- |
| Intercept + 1+2+3+4 | 2.52 | 0.93 |
| Intercept + 1+2+3 | 1.06 | 0.74 |
| Intercept + 1+2+4 | 0.96 | 0.72 |
| Intercept + 1+3+4 | 0.78 | 0.69 |
| Intercept + 2+3+4 | 0.11 | 0.53 |
| Intercept + 1+2 | -0.50 | 0.38 |
| Intercept + 1+3 | -0.67 | 0.34 |
| Intercept + 1+4 | -0.78 | 0.31 |
| Intercept + 2+3 | -1.34 | 0.21 |
| Intercept + 2+4 | -1.45 | 0.19 |
| Intercept + 3+4 | -1.63 | 0.16 |
| Intercept + 1 | -2.24 | 0.10 |
| Intercept + 2 | -2.90 | 0.05 |
| Intercept + 3 | -3.08 | 0.044 |
| Intercept + 4 | -3.19 | 0.040 |
| Intercept | -4.64 | 0.01 |

**Supplementary Data S5**. Performance of the definition developed using the multivariable logistic regression model in the internal-external cross-validation.


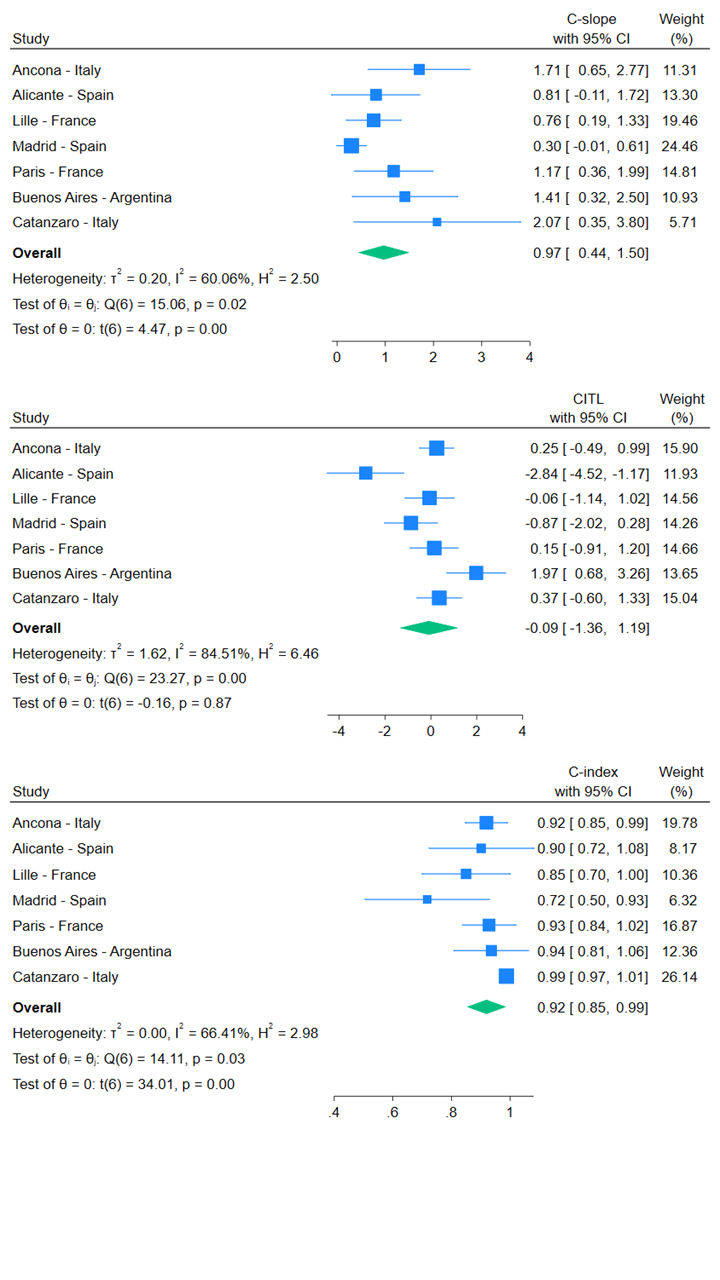


**Supplementary Data S6**. Discrimination of the proposed patient-reported definitions for acute CPP crystal arthritis across sensitivity analyses.

| **Definitions** | **Sensitivity**  **(95%CI)** | **Specificity**  **(95%CI)** | **C-index**  **(95%CI)** |  |
| --- | --- | --- | --- | --- |
| **Patients with at least one episode of acute CPP crystal arthritis in their medical history [N=205]** | | | |  |
| CART | 0.85 (0.75 to 0.92) | 0.83 (0.76 to 0.89) | 0.84 (0.79 to 0.89) |  |
| Multivariable logistic regression model | 0.84 (0.74 to 0.91) | 0.85 (0.78 to 0.91) | 0.84 (0.79 to 0.89) |  |
| **Patients without any episode of acute CPP crystal arthritis in their medical history [N=41]** | | | |  |
| CART | 0.77 (0.50 to 0.93) | 0.83 (0.63 to 0.95) | 0.80 (0.67 to 0.93) |  |
| Multivariable logistic regression model | 0.82 (0.57 to 0.96) | 0.92 (0.73 to 0.99) | 0.87 (0.76 to 0.98) |  |
| **Female sex [N=162]** | | | |  |
| CART | 0.86 (0.75 to 0.94) | 0.86 (0.78 to 0.92) | 0.86 (0.81 to 0.92) |  |
| Multivariable logistic regression model | 0.90 (0.79 to 0.96) | 0.86 0.78 to 0.92) | 0.88 (0.83 to 0.93) |  |
| **Male sex [N=84]** | | | |  |
| CART | 0.78 (0.62 to 0.90) | 0.77 (0.62 to 0.88) | 0.78 (0.68 to 0.87) |  |
| Multivariable logistic regression model | 0.73 (0.56 to 0.86) | 0.85 (0.72 to 0.94) | 0.79 (0.70 to 0.88) |  |
| **Included blood inflammatory markers in the model [N=246]** | | | |  |
| CART | 0.83 (0.74 to 0.90) | 0.83 (0.76 to 0.88) | 0.83 (0.79 to 0.88) |  |
| Multivariable logistic regression model | 0.83 (0.74 to 0.90) | 0.86 (0.79 to 0.91) | 0.85 (0.80 to 0.89) | |
| **Replaced patient-reported swelling and warmth with physician-evaluated outcomes [N=246]** | | | |  |
| CART | 0.78 (0.69 to 0.86) | 0.85 (0.79 to 0.91) | 0.82 (0.77 to 0.87) |  |
| Multivariable logistic regression model | 0.83 (0.74 to 0.90) | 0.86 (0.79 to 0.91) | 0.85 (0.80 to 0.89) |  |
| **The target joint was the knee [N=119]** | | | |  |
| CART | 0.79 (0.64 to 0.89) | 0.79 (0.68 to 0.88) | 0.79 (0.71 to 0.87)1 |  |
| Multivariable logistic regression model | 0.75 (0.60 to 0.86) | 0.82 (0.71 to 0.90) | 0.78 (0.71 to 0.86) |  |
| **The target joint was a joint other than the knee [N=127]** | | | |  |
| CART | 0.88 (0.75 to 0.95) | 0.87 (0.78 to 0.94) | 0.88 (0.82 to 0.93) |  |
| Multivariable logistic regression model | 0.92 (0.80 to 0.98) | 0.90 (0.81 to 0.96) | 0.91 (0.86 to 0.96) |  |
| **Including the pain at rest >1 as an absolute inclusion criterion [N=246]** | | | |  |
| CART | 0.80 (0.71 to 0.88) | 0.83 (0.76 to 0.89) | 0.82 (0.77 to 0.87) |  |
| Multivariable logistic regression model | 0.80 (0.71 to 0.88) | 0.87 (0.80 to 0.92) | 0.83 (0.79 to 0.88) |  |
| **Including the pain at rest >2 as an absolute inclusion criterion [N=246]** | | | |  |
| CART | 0.76 (0.66-0.84) | 0.86 (0.80-0.91) | 0.81 (0.76-0.86) |  |
| Multivariable logistic regression model | 0.77 (0.67-0.85) | 0.87 (0.81-0.92) | 0.82 (0.77-0.87) |  |
| **Including the pain at rest >3 as an absolute inclusion criterion [N=246]** | | | |  |
| CART | 0.71 (0.61-0.80) | 0.87 (0.81-0.92) | 0.79 (0.74-0.84) |  |
| Multivariable logistic regression model | 0.70 (0.60-0.79) | 0.88 (0.82-0.93) | 0.79 (0.74-0.84) |  |
| **Including the pain at rest >4 as an absolute inclusion criterion [N=246]** | | | |  |
| CART | 0.60 (0.50-0.70) | 0.89 (0.83-0.93) | 0.75 (0.70-0.81) |  |
| Multivariable logistic regression model | 0.58 (0.48-0.68) | 0.90 (0.84-0.94) | 0.74 (0.69-0.80) |  |
| **Including the pain at rest >5 as an absolute inclusion criterion [N=246]** | | | |  |
| CART | 0.54 (0.44-0.64) | 0.91 (0.86-0.96) | 0.73 (0.68-0.79) |  |
| Multivariable logistic regression model | 0.52 (0.42-0.62) | 0.91 (0.85-0.95) | 0.71 (0.66-0.77) |  |
| 95%CI: 95% confidence interval, CART: classification and regression tree, CPP: calcium pyrophosphate. | | | |  |

**Supplementary Data S7**. Discrimination of the proposed definitions across participating centres.

**Definitions based on multivariable logistic model**

**
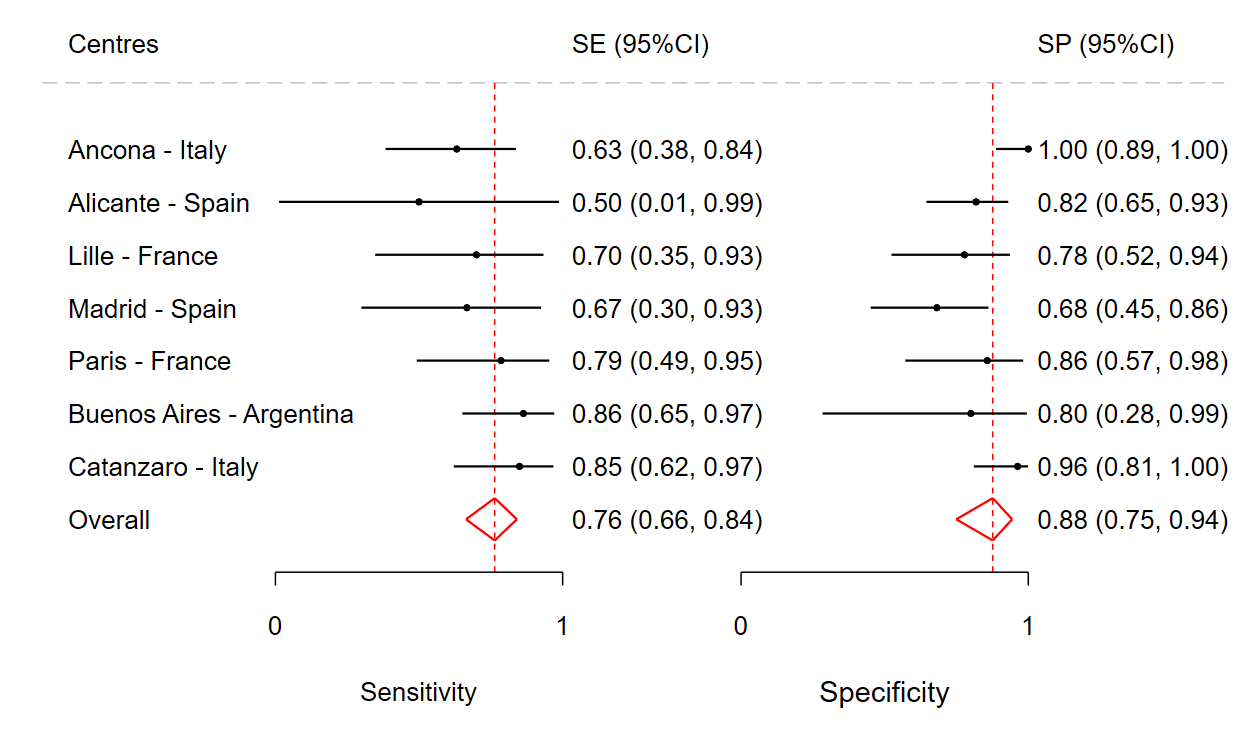
**

**Definitions based on classification and regression tree analysis**

**
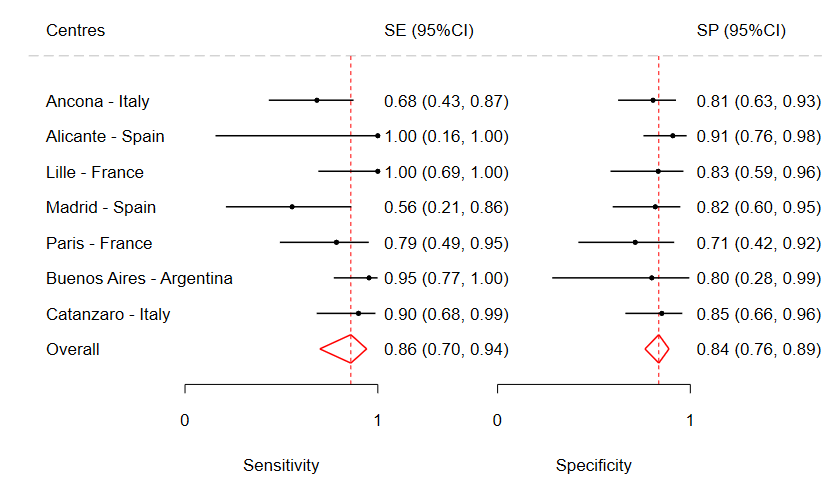
**

**SE**: sensitivity, **SP**: specificity

**Supplementary Data S8**. Final equation after internal validation

Probability of having acute CPP crystal arthritis = $\frac{e^{lp}}{{1+e}^{lp}}$

| Where lp = | - 4. 6417817 |
| --- | --- |
|  | + 2.4070697 * patient-defined flare (yes=1, no=0) |
|  | + 1.7376265 * patient-defined joint swelling (yes=1, no=0) |
|  | + 1.5609486 * patient-defined joint warmth heating (yes=1, no=0) |
|  | + 1.4536605 * time from the onset to the peak pain (<48h=1, >48h=0) |
